# The pathogenesis of liver cancer and the therapeutic potential of bioactive substances

**DOI:** 10.3389/fphar.2022.1029601

**Published:** 2022-10-05

**Authors:** Song Gao, Xingyue Jiang, Liang Wang, Shanshan Jiang, Hanyuan Luo, Yan Chen, Cheng Peng

**Affiliations:** ^1^ State Key Laboratory of Southwestern Chinese Medicine Resources, Chengdu, China; ^2^ School of Public Health, Chengdu University of Traditional Chinese Medicine, Chengdu, China; ^3^ College of Pharmacy, Chengdu University of Traditional Chinese Medicine, Chengdu, China

**Keywords:** liver cancer, pathogenic factors, bioactive substances, mechanisms of action, bioavailability

## Abstract

Liver cancer is the third most common cause of cancer-related deaths in the world and has become an urgent problem for global public health. Bioactive substances are widely used for the treatment of liver cancer due to their widespread availability and reduced side effects. This review summarizes the main pathogenic factors involved in the development of liver cancer, including metabolic fatty liver disease, viral infection, and alcoholic cirrhosis, and focuses on the mechanism of action of bioactive components such as polysaccharides, alkaloids, phenols, peptides, and active bacteria/fungi. In addition, we also summarize transformation methods, combined therapy and modification of bioactive substances to improve the treatment efficiency against liver cancer, highlighting new ideas in this field.

## 1 Introduction

Liver cancer is the third most common cause of cancer-related deaths in the world (Sällberg and Pasetto, 2020) and is divided into primary and secondary liver cancer. Primary liver cancer includes hepatocellular carcinoma (HCC), cholangiocarcinoma and mixed liver cancer. Secondary liver cancer is when the primary tumor originates in other parts of the body and then metastasizes to the liver through the blood or other routes. This includes liver metastasis from intestinal cancer, breast cancer and others. Besides, primary lymphoma, hepatoblastoma, angiomyolipoma, etc. Are also common malignant tumors of the liver. Studies have shown that the estimated incidence of liver cancer will exceed 1 million cases by 2025, with HCC being the most common form, accounting for 90% of all cases. This condition is a significant economic and public health burden for the whole world (Llovet, 2003). Therefore, it is urgent to find more efficient and economical treatments.

There are some similarities in the pathogenesis of the various forms of liver cancer. The main risk factors are long-term infection with hepatitis B virus (HBV), hepatitis C virus (HCV), hepatitis D virus (HDV), alcoholic cirrhosis, nonalcoholic steatohepatitis (NASH), obesity/type 2 diabetes, autoimmune hepatitis, and eating aflatoxin B1-contaminated food (Gingold et al., 2018). In addition, the geographic region, gender, and age may also be related to the occurrence of liver cancer (Qiu et al., 2019; Sayiner et al., 2019).

There are various treatment methods for liver cancer, but the main treatment method at present is still surgical treatment. Even if the liver cancer is radically removed, there are still problems such as postoperative liver cancer metastasis, high recurrence rate and poor prognosis. In addition, surgery has caused certain psychological and physical trauma to patients, which not only reduces the patient’s living index, but also poses a huge challenge to prolonging the survival period of liver cancer patients (Man et al., 2021). For advanced HCC patients who are not suitable for transplantation or fail local and regional therapy, first-line drugs such as sorafenib and lenvatinib are more clinically selected (Pinter et al., 2009), but their use is hindered by drug resistance. Studies have shown that sorafenib is only effective in 35%–43% of patients, and most patients have disease progression within 6 months, along with side effects of diarrhea and skin reactions on the hands and feet (Dika and Abou-Alfa., 2017). Therefore, exploring a therapeutic approach with curative effect on liver cancer and low side effects has become a new direction for cancer treatment in the future.

At present, bioactive substances have been very eye-catching in the treatment of liver cancer. Of a total of 185 small molecule anti-cancer compounds approved for clinical use, only 29 (15.7 percent) are reported to be classified as fully synthetic, with the remaining 156 (84.3 percent) being either natural products themselves or inspired by natural products (Newman and Cragg, 2020). Biologically active substances have the characteristics of diverse structure, low toxicity and wide range of sources, and have unique advantages and great potential in the treatment of liver disease (Shan et al., 2019). Many studies have shown that many bioactive substances such as paclitaxel and curcumin have significant anti-tumor efficacy and have fewer side effects than other chemotherapy drugs (Choudhari et al., 2020). In addition, some bioactive substances can not only enhance the cytotoxicity of chemotherapy drugs in cancer cells, but also protect healthy cells from adverse reactions caused by chemotherapy drugs through various pathways such as antioxidant, anti-inflammatory, and anti-apoptosis (Arora et al., 2019; Abd Rashid et al., 2021). Therefore, exploring the mechanism of biologically active substances in the treatment of liver cancer is of great significance for the development of the field of cancer treatment. At present, the phase I human clinical trial of urtic acid nanolipids (CTR20160681) and the phase I clinical trial of chlorogenic acid for injection in subjects with advanced cancer (CTR20130586) of phase I clinical trials (CTR20130586) have been approved for clinical trials. In this review, we focus especially on the therapeutic mechanisms by which bioactive substances (such as polysaccharides, alkaloids, phenols, peptides, and active bacteria/fungi) target liver cancer in the preclinical stage. We highlight the advantages brought by these active substances, aiming to provide new ideas for the treatment of liver cancer and further promote bioactive substances into clinical trials.

## 2 Pathogenic factors

### 2.1 Metabolic fatty liver disease

Nonalcoholic fatty liver disease (NAFLD) (which in 2020 an international panel of experts recommended to be renamed metabolic dysfunction associated fatty liver disease (MAFLD)) (Eslam et al., 2020) is a well-known risk factor for HCC (Yamamoto et al., 2021) and encompasses a range of pathological changes, including steatosis, NASH, fibrosis, and cirrhosis (Samuel and Shulman, 2018). Its global incidence rate is about 25.2% in the adult population, and has become the most common chronic liver disease. Because of its high prevalence rate, nonalcoholic fatty liver disease is now the fastest-growing cause of liver-related mortality in the world, and it is becoming an important cause of end-stage liver disease, including primary liver cancer. A frequent outcome of this condition is liver transplantation. Approximately 3%–15% of obese patients with NASH develop cirrhosis each year, and 2.4%–12.8% of patients with non-alcoholic liver cirrhosis develop primary liver cancer (mainly HCC). However, in the absence of cirrhosis, patients with NASH can also develop HCC from the beginning. Approximately 4%–27% of NASH patients with cirrhosis develop HCC (Dhamija et al., 2019; Selby et al., 2020). In view of the high prevalence of obesity-related nonalcoholic fatty liver disease, many experts predict that nonalcoholic fatty liver disease will soon become the main cause of HCC, especially in the United States and other Western countries (Sayiner et al., 2019).

**FIGURE 1:**
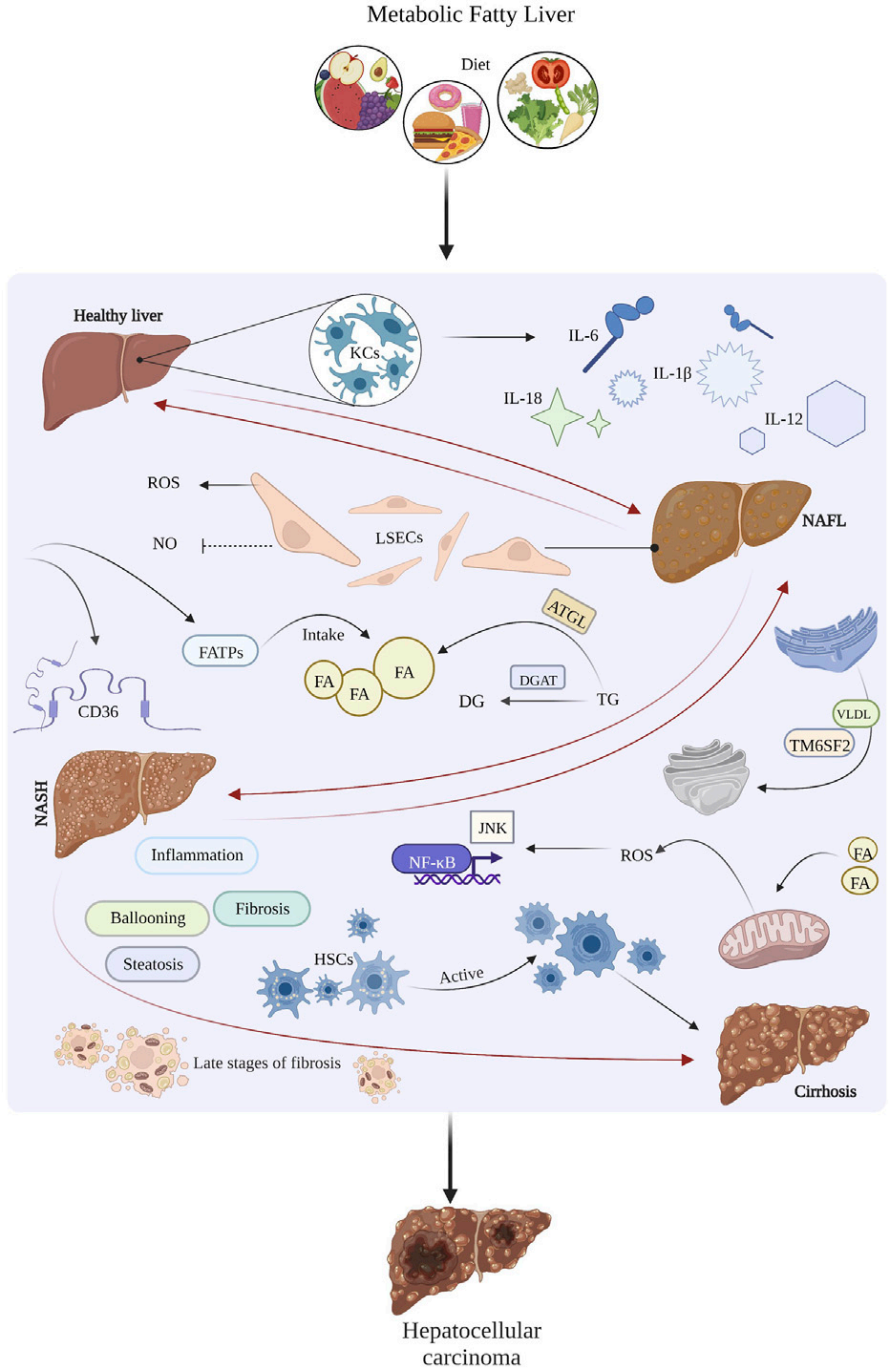
The main mechanism of hepatic carcinoma caused by metabolic fatty liver disease.

Nonalcoholic fatty liver disease is currently the second leading cause of end-stage liver disease and the second most common cause of primary liver cancer in adults awaiting liver transplantation in the United States (Powell et al., 2021). NAFLD is associated with 14.1% of HCC cases, and the incidence of NAFLD-related HCC is increasing by 9% per year (Kanda et al., 2020). A recent modelling study estimated that by 2030, the prevalence of NASH will increase by 63% in the United States, resulting in a 168% increase in NASH-related decompensated cirrhosis, a 137% increase in HCC, a 178% increase in liver-related death, and an estimated 800,000 excess liver deaths (Younossi et al., 2019). In Latin America, MAFLD affects 31% of the population, including 35.2% in Brazil, 23% in Chile, 17% in Mexico, and 26.6% in Colombia (Méndez-Sánchez and Diaz-Orozco., 2021). Also in Europe, non-alcoholic fatty liver disease now accounts for 8.4% of all transplants performed annually, and among all recipients of liver transplants, hepatocellular carcinoma was found more frequently in those with non-alcoholic fatty liver disease (39.1%) than in those without it (28.9%) (Powell et al., 2021). A study in Britain showed that one of every three HCC patients had NASH, indicating that NAFLD may have become the main cause of HCC in that country (Augustin et al., 2017). The main mechanisms underlying the development of liver cancer in metabolic fatty liver disease are shown in Figure 1.

### 2.2 Viral infection

**FIGURE 2:**
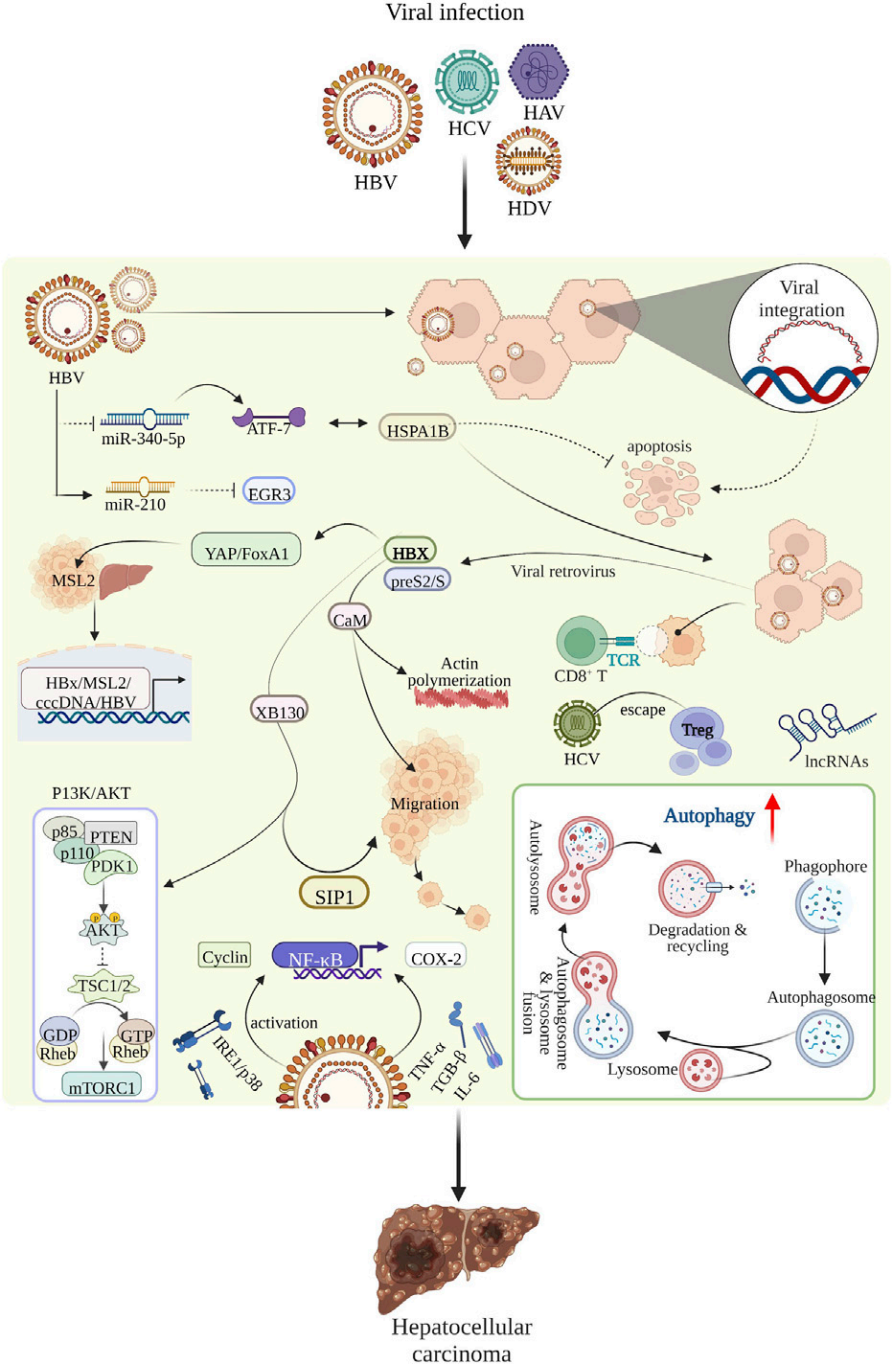
The main mechanism of viral infection-induced hepatocellular carcinoma.

A risk factor for primary liver cancer is chronic inflammation leading to chronic necrotizing inflammation (Kanwal et al., 2018). This is caused by viral infections, including HBV, HCV, HDV and other factors (Anwanwan et al., 2020). Among these, hepatitis B is the most prevalent form of viral hepatitis in the world (Lin et al., 2020). The mechanisms underlying hepatitis virus-induced liver cancer include integration of hepatitis B virus DNA into the host cell genome, metabolic reprogramming due to viral infection (Tu et al., 2017), the immune response, viral proteins affecting signaling pathways, cellular stress response pathways and inflammatory responses. The main mechanisms underlying viral-induced liver cancer are shown in Figure 2.

#### 2.2.1 Gene regulation

HBV infects and integrates only into hepatocytes (Chen et al., 2020) and is the only hepadnavirus that utilizes viral DNA integration to induce genomic instability. This can lead to the production of fusion gene products and alter the expression of oncogenes or tumor suppressors. HCV does not encode oncoproteins or integrate its genome into host chromosomal DNA. The mechanism underlying HCV-associated HCC carcinogenesis is mainly indirect, due to viral regulation of host cellular processes, resulting in hepatocyte steatosis, inflammation, oxidative stress, immune responses, etc. HDV can co-infect with HBV or be acquired through superinfection by horizontal transmission in chronic hepatitis B patients (Ringelhan et al., 2017; D’souza et al., 2020).

MicroRNAs (miRNAs) are a class of short endogenous non-coding RNAs that bind to target mRNAs and inhibit their expression through sequence-dependent mechanisms and play an important role in gene regulation (Ghafouri-Fard et al., 2021). Multiple studies have confirmed that miR-221, miR-210, miR-223, miR-21, miR-155, miR-455, as well as miR-145 and miR-194 are involved in HCC (Song et al., 2019). miR-148a, miR-602, miR-143, miR29a, miR-152, miR-373, miR-16, miR-101, and miR-661 are associated with HBV-mediated HCC (Chaturvedi et al., 2019). miR-340-5p is a miRNA identified as a tumor suppressor gene. HBV can enhance the expression of activating transcription factor 7 (ATF7) by downregulating miR-340-5p, which in turn interacts with heat shock protein family A member 1B (HSPA1B), promoting cell proliferation and inhibiting apoptosis, which in turn affects the development of liver cancer (Ghafouri-Fard et al., 2021). In liver tissue with HBV-associated cirrhosis and HCC, the expression of microRNA-210 (miRNA-210) is increased, and the content of EGR3 is decreased. Li et al. confirmed that silencing miRNA-210 can inhibit HepG2 by up-regulating EGR3 to promote apoptosis (Li X. et al., 2020). In tissues with HBV-associated HCC, miR-1271-5p was down-regulated and AQP5 was up-regulated (Li Z. et al., 2021). miR-122 can specifically bind to the 3′-untranslated region (3′UTR) of apolipoprotein B mRNA editing enzyme catalytic subunit 2 (APOBEC2) mRNA and inhibit its expression. miR-122 targets the 3′UTR of APOBEC2 mRNA and can induce HCC (Li et al., 2019).

HBV covalently closed circular DNA (cccDNA) is critical in the development of hepatocellular carcinoma. The integration of HBV DNA into host hepatocyte genes plays a key role in reverse transcription and replication of the virus and in the production of HBV X (HBx) or preS2/S proteins, which contribute to tumorigenesis by interfering with gene expression or activating oncogenic signaling pathways (Chaturvedi et al., 2019). Feng et al. showed that long non-coding RNA (lncRNA) PCNAP1 enhanced HBV replication by regulating the miR-154/PCNA/HBVcccDNA signaling pathway. PCNAP1 enhanced PCNA by acting as a sponge for miR-154, targeting the PCNA mRNA 3′UTR and PCNAP1/PCNA signaling pathway to drive hepatocarcinogenesis (Feng J. et al., 2019). In addition, transforming growth factor beta-activated long non-coding RNA (lncRNA-ATB), a novel oncogenic lncRNA stimulated by transforming growth factor β, was increased in advanced metastatic tumors and liver cancer tissues, and was also significantly associated with HBV infection (Zhang Y. et al., 2020).

#### 2.2.2 Immunomodulation

Chronic infection with hepatitis viruses is a major risk factor for liver injury and HCC. In chronic viral hepatitis, the development of fibrosis, cirrhosis, and HCC is associated with the immune response against the infected liver (Zambam de Mattos et al., 2021). Disturbances in the function of virus-specific T cells are a major factor underlying the incapacity of the immune response to clear infected cells on the one hand, and on the other hand, it influences the development of the disease (Healy et al., 2020). Cytotoxic CD8^+^ T cells are key immune effectors against tumors. The mechanism by which immune cells promote HCC is mainly through the secretion of cytokines and growth factors, which enhance the proliferation and inhibit the apoptosis of tumor cells (Llovet, 2003).

Autophagy, an important defense and protection mechanism ubiquitous in eukaryotic cells, is related to the body’s innate and adaptive immunity and plays a key role in the regulation of liver physiology and homeostasis. Impaired autophagy can explain the pathogenesis of various liver diseases, such as hepatitis. The role of autophagy in liver cancer is dual. In the early stage of tumorigenesis, autophagy acts as a tumor suppressor, preventing genomic instability by removing damaged organelles and proteins; in later stages of tumor development, transformed cancerous and stromal cells utilize autophagy to produce nutrients in the tumor microenvironment, promoting the progression and progression of established liver tumors. In addition, autophagy in tumor cells and the host, as well as in the surrounding microenvironment, promotes tumorigenesis and cancer development (Chao et al., 2020).

In chronic hepatitis caused by viruses, T cells are continuously stimulated by the persistence of viral antigens. Programmed cell death protein 1 (PD-1) and CD4^+^Foxp3^+^ regulatory T cells (Tregs) are immune activating and suppressive factors which play an important role in maintaining the balance. HBV is genetically stable but its viral antigens persist, leading to immune activation followed by loss of energy or dysfunction. HBV surface antigen (HBsAg) particles are overproduced and secreted, and viral particles whose surface is mainly composed of PreS1 and PreS2 can escape from antibodies and infect new cells. While HCV is constantly changing, it persists by evading B and T cells (Sällberg and Pasetto, 2020).

#### 2.2.3 Regulation of signaling pathways by viral proteins

The regulation of cellular signaling pathways by viral proteins is one of the mechanisms by which HBV, HCV and HDV drive hepatocarcinogenesis (D’souza et al., 2020). HBx, one of the HBV gene products, plays a key role in the occurrence and metastasis of liver cancer. HBx can induce the up-regulation of male-specific lethal (MSL2) in stably transfected HBx hepatoma cell lines and HBx transgenic mice by activating yes-associated protein (Yap)/FoxA1 signaling, forming a positive feedback loop (HBx/MSL2/cccDNA/HBV) that regulates HBV cccDNA and promotes hepatocarcinogenesis (Gao et al., 2017). HBx promotes actin polymerization and hepatoma cell migration by regulating the protein calmodulin (CaM) (Kim et al., 2021). Zhou et al. proposed that the HBV X protein captures protons and chloride ions and induces collagen expression in the liver, forming strong hydrogen bonds with the captured protons, and that the HBV X protein and collagen locally accumulate HCl, triggering cirrhosis in some patients The disease progresses to liver cancer (Zhou et al., 2017). HBx can modulate biological processes in HepG2 cells and accelerate the development of HCC by activating the XB130-mediated phosphatidylinositol 3-kinase (PI3K)/AKT pathway (Huang et al., 2021). HBx increases the expression of Smad-interacting protein 1 (SIP1) and recruits it to the promoter of E-cadherin to repress the transcription of E-cadherin, and the consequent decrease in E-cadherin expression blocks intercellular adhesion and attachment, thereby promoting tumor invasiveness (Ye et al., 2019). After HBx vector transfection, the expression levels of lncRNA-ATB and transforming growth factor-β (TGF-β) increased in HepG2 cells, accompanied by increased autophagy, which may be a potential mechanism by which HBV can induce HCC (Zhang Y. et al., 2020).

Large HBV surface proteins (LHBs) contain an intact pre-S1 domain that binds to the HBV receptor sodium taurocholate cotransport polypeptide on hepatocytes to facilitate viral entry. Pre-S2 mutant LHBs are important viral oncoproteins. For example, the pre-S2 variant surface protein binds to JAB1 and induces nuclear translocation of JAB1, thereby activating the p27/retinoblastoma/Cdk2/cyclin A pathway and promoting cell cycle progression (Lin et al., 2020).

#### 2.2.4 Oxidative stress and endoplasmic reticulum stress

Oxidative stress and endoplasmic reticulum stress (ERs) have been observed in chronic inflammatory liver disease induced by hepatoviral infection and with oncogenic potential, resulting in the production of a large number of cytokines and chemokines. Endoplasmic reticulum stress in macrophages activates the pro-inflammatory GSK-3β, nuclear factor kappa B (NF-κB) and mitogen-activated protein kinase (MAPK) pathways, and stress signals can propagate to individual hepatocytes through potent mediators. This activates the unfolded protein-mediated cell death response in nearby non-parenchymal cells (Li X. et al., 2021). Expression of viral LHBs in chronic HBV carriers also triggers a sustained ER overload response, leading to activation of ER stress signaling pathways. Pre-S mutant-induced protein misfolding triggers the unfolded protein response (UPR) and endoplasmic reticulum stress-mediated signaling, including IRE1/p38-mediated activation of NF-κB and COX-2, calcium efflux, calpain cleavage and cyclin A overexpression, leading to centrosome overduplication (Lin et al., 2020).

#### 2.2.5 Inflammation

HCC is a well-known example of a tumor induced by inflammation, and a variety of immune and inflammatory factors, including T cells, cytokines, etc., play crucial roles in the development of HCC, including interleukin-6 (IL-6), interferon-α (TNF-α), TGF-β, and other cytokines (Chaturvedi et al., 2019). HBV-related HCC is induced mainly by the host’s immune and inflammatory responses (Yuan et al., 2021), and oncogenic transformation in related cancers is often accompanied by long-term inflammation and cirrhosis (Chaturvedi et al., 2019). Serum IL-6 and interleukin-1β (IL-1β) levels are abnormally elevated in HCC patients, and there are many collagen fibers in liver cancer tissue. Interfering with c-X-c motif chemokine receptor 3 (cXcr3) can inhibit cell proliferation and migration. It can also reduce the expression levels of the α1 chain of collagen type I and proteins in the Tlrs/Myd88 pathway, promoting apoptosis in HBV-lX-2 cells (Yuan et al., 2021).

### 2.3 Alcohol

The liver is the main organ involved in alcohol metabolism (Hyun et al., 2021). Excessive alcohol consumption can result in fatty liver, acute/chronic hepatitis, liver fibrosis and cirrhosis, ultimately leading to HCC (Seitz et al., 2018). Studies have shown that approximately 154,700 cases of hepatocellular carcinoma in 2020 were associated with alcohol consumption (Rumgay et al., 2021b). Related studies have also found that there is a linear dose-response relationship between alcohol consumption and the risk of cirrhosis and HCC. The odds ratio (OR) value for cirrhosis increased when alcohol intake was 30–50 g/day, while the OR value for HCC increased when alcohol intake was >60–100 g/day (Matsushita and Takaki, 2019). A World Cancer Research Fund (WCRF) analysis showed that for every 10 g of alcohol consumed per day, the risk of HCC increases by 14% (RR 1.14 (95% CI 1.04–1.25)) (Rumgay et al., 2021a).

Underlying alcohol-induced liver cancer is the process of alcohol metabolism. Ethanol is first oxidatively degraded into acetaldehyde by alcohol dehydrogenase (ADH) in the cytoplasm of hepatocytes, and then acetaldehyde is oxidized to non-toxic acetic acid by acetaldehyde dehydrogenase 2 (ALDH2) and coenzyme NAD or NADP, for excretion (Wang W. et al., 2020). Under conditions of excessive ethanol intake, cytochrome P450 2E1 (CYP2E1) in the endoplasmic reticulum and catalase in peroxisomes also become involved in the metabolism of ethanol, catalyzing the conversion of ethanol into acetaldehyde, and generating reactive oxygen species (ROS) (Matsushita and Takaki, 2019). Therefore, excess ethanol will generate toxic by-products such as acetaldehyde and ROS, which accumulate in the liver. Acetaldehyde interferes with DNA repair and promotes lipid peroxidation, adduct formation, mitochondrial damage and DNA mutagenesis, ultimately contributing to hepatocarcinogenesis (Taniai, 2020). At the same time, accumulation of ROS contributes to the development of HCC by inducing DNA damage, genomic vulnerability in hepatocytes and suppressing T lymphocytes (Ogunwobi et al., 2019).

Interference with normal immune system function is another important mechanism of alcohol-induced liver cancer. Chronic alcohol consumption can impair immune system function and exacerbate infections (Little et al., 2019). Natural killer cells (NK) make up 30%–50% of liver lymphocytes and play an important role in tumor surveillance (Male et al., 2017). Long-term alcohol consumption may cause NK cell dysfunction. Alcohol intake not only decreases the cytotoxic function of NK cells, but also decreases the number of NK cells (Male et al., 2017; Matsushita and Takaki, 2019). Yan et al. investigated the immunological mechanisms underlying ethanol-associated tumor progression and found that ethanol not only aggravated liver tumor progression, but also decreased the number of antitumor CD8^+^ T cells (Yan et al., 2017). Furthermore, hepatic stellate cell-macrophage interactions may contribute to the induction of myeloid-derived suppressor cells (MDSCs), thereby inhibiting tumor surveillance by immune cells and promoting a tumorigenic microenvironment in the early and late stages of HCC (Matsuda and Seki, 2020).

**FIGURE 3:**
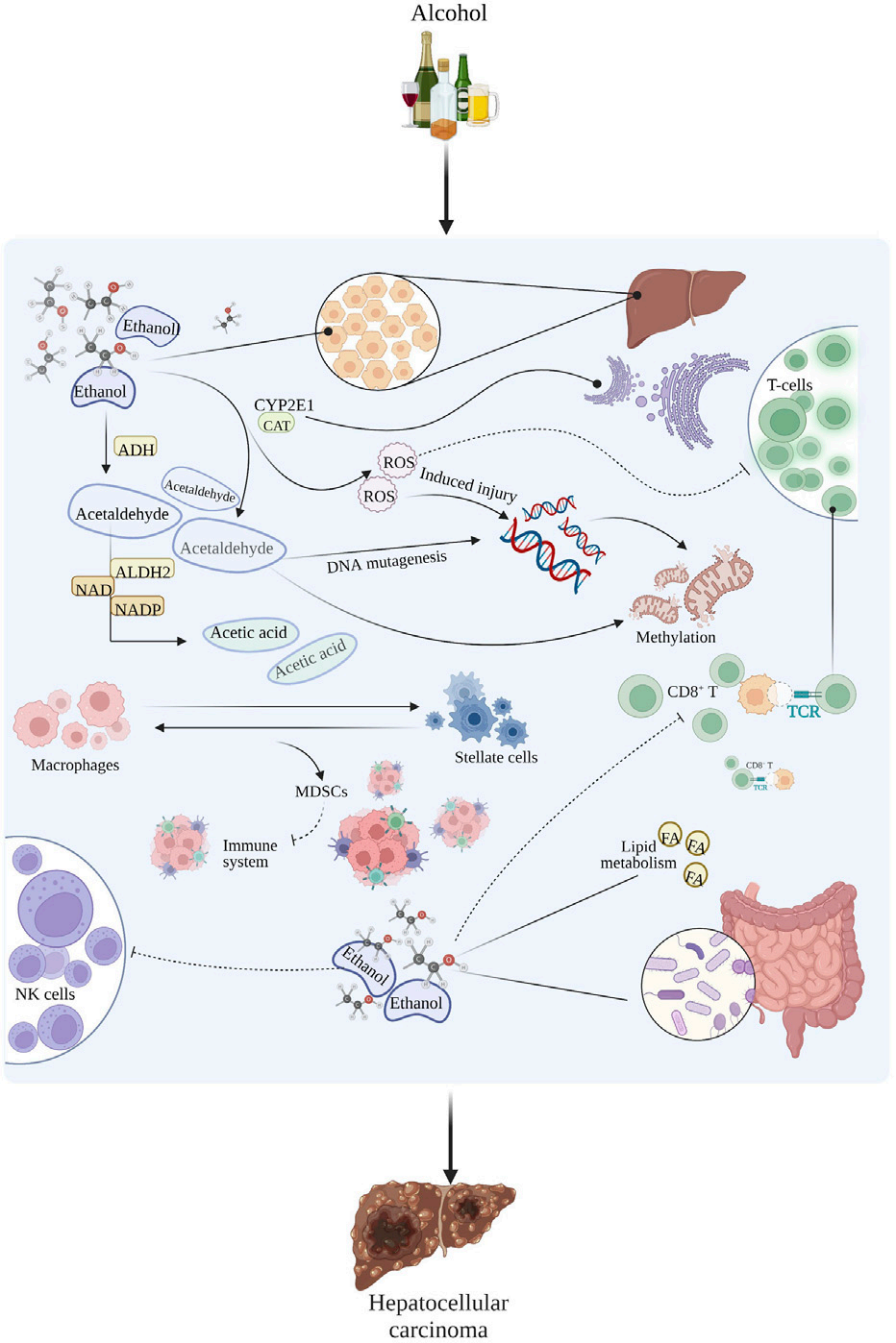
The main mechanism of alcohol-induced liver cancer.

In addition to the two previously mentioned mechanisms, alcohol-induced liver cancer may also be closely related to lipid metabolism (Ganne-Carrié and Nahon, 2019), intestinal flora disturbances (Akkız, 2021), DNA methylation (Udali et al., 2015), and others. The main mechanisms underlying alcohol-induced liver cancer are shown in Figure 3.

## 3 Therapeutic effect of bioactive substances on liver cancer

**FIGURE 4:**
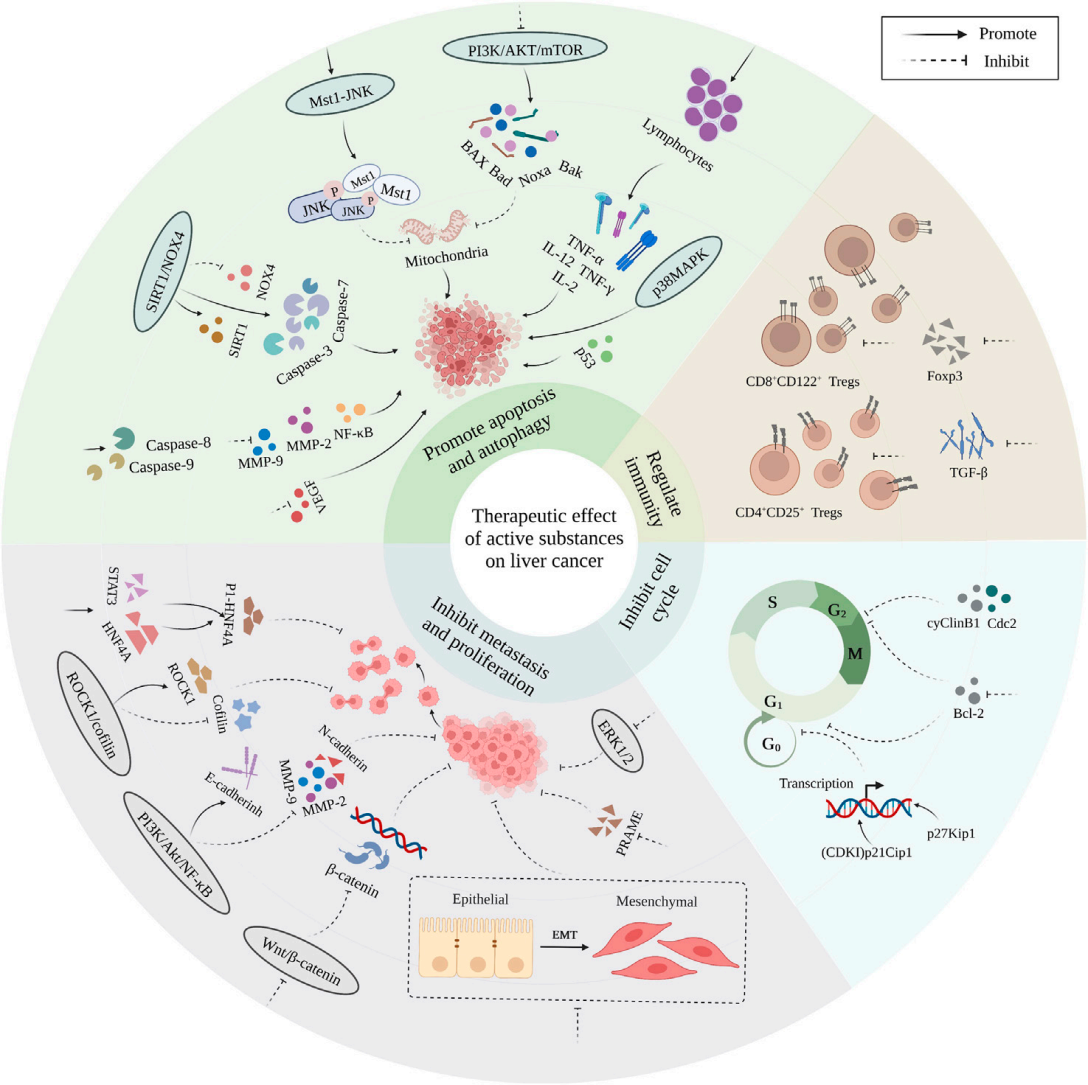
Diagram of the main mechanism of bioactive substances in the treatment of liver cancer.

Polysaccharides, alkaloids, phenols, polypeptides, and active bacteria/fungi are the main bioactive substances showing therapeutic potential against liver cancer. Bioactive substances can play a positive role in the treatment of liver cancer through the following four pathways: 1. By regulating related signaling pathways, inducing apoptosis and autophagy; 2. By inhibiting the proliferation and migration of liver cancer cells; 3. By regulating the cell cycle and inhibiting the growth of liver cancer cells; 4. By regulating immunity. The main mechanisms underlying the therapeutic effects of bioactive substances against liver cancer are shown in Figure 4 and Table 1.

**TABLE 1:** Anti-hepatoma mechanism/effect table of active substances (↑ increase/enhance, ↓ decrease/inhibit).

Kind	Active substance	Mechanism/Effect	Research model	References
Polysaccharides	*Dandelion* polysaccharide	↓The protein/mRNA expression level of Hepc; ↓JAK/STAT	HepG2, Huh7, Hepa1-6, H22 cells	Ren et al. (2021a)
	Basil polysaccharide	↑E-Cadherin/VMP1 mRNA/protein expression level; The mRNA/protein expression level of N-cadherin/*Vimentin*↓; ↓ β-catenin	MHCC97H cells, MHCC97L cells	Feng et al. (2019a)
	Alkaline-soluble polysaccharide/water-soluble polysaccharide	Phagocytosis of macrophages↑; IL-2, IL-12, TNF-γ, TNF-α↑	PC3 human prostate cancer cells, HepG2, MCF-7 human breast cancer cells	Nazeam et al. (2017)
	*Sipunculus nudus* polysaccharide	↑IL-2, IFN-γ, TNF-α; ↑DDIT3; ↓Cyr61, Hsp90, Gene expression level of VEGF; ↑caspase-3, caspase-8, caspase-9, Bax	Human hepatocyte cells (LO2), Male athymic NU/NU mice	Su et al. (2021)
	Exopolysaccharide 11	Migration of cancer cells↓; ↓The mRNA/protein expression level of CD99	Huh7.5, HepG2 and Bel-7402 liver cancer cells, B16F-10 cells	(Wang et al., 2019d; Liu et al., 2021a)
	Exopolysaccharides 364	↓FGF19-FGFR4 signaling axis; ↑ROS; ↓Growth and adhesion of cancer cells	Huh7.5, HL-7702, Bel-7402 cells	Wang et al. (2021a)
	*N*-dihydrogalactochitosan	↑Sensitivity of tumor cells to radiation	4T1 triple-negative murine breast cancer cells	Wang et al. (2019a)
	Water-soluble yeast β-D-glucan	↑PH of lysosome; ↓Cathepsin activity of lysosome; ↑ROS	Huh7, SMMC-7721, LM3, HL-7702 cells	Wang et al. (2020a)
	*Ganoderma lucidum* polysaccharide	↑M1 macrophage; ↓M2 macrophage; PI3K/AKT pathway↓; ↑ Bax, Bad, Bak, Noxa	Mouse H22 cells	Song et al. (2021)
	*Dictyophora* polysaccharide	Hepatocellular carcinoma cell growth↓	HCC-LM3 human hepatocellular carcinoma cells	Song et al. (2021)
Alkaloids	Berberine	By↓GPT1, ↓Hepatocellular carcinoma cell growth	PLC/PRF/5 cell, MHCC97L cells tagged with luciferase gene, normal human liver MIHA cells	Guo et al. (2020b)
		↓Akt pathway, ↓Skp2 expression, ↑FOXO3a expression, ↑Transcription of p21^Cip1^ and p27^Kip1^	Human hepatoma cell line Huh-7 and HepG2	Li et al. (2018)
		↑67LR, ↑cGMP, ↑caspase-8/3	SMMC7721, HepG2, BEL7402, LO2, H9	Zhou et al. (2018)
	Tetrandrine	↓EMT, ↑p-β-catenin, ↓Wnt/β-catenin signaling pathway	Human hepatoma cell line Huh7 and Hep3B, cell lines HCCLM9	Zhang et al. (2018b)
		↓p-CaMKIIδ	HuH7, SMMC-7721, HepG2, PLC/PRF/5, SK-Hep-1, SNU398, MHCC97H cell	Huang et al. (2019)
	Lycorine	↓cyclin A, ↓cyclin B1, ↓cdc2, ↓ROCK1/cofilin	Human hepatoblastoma cell line HepG2	Liu et al. (2019)
		↓CDK1	Human hepatoma cell line HepG2, HuH-7 and Hep3B	Yin et al. (2021)
	Solamargine	↓LIF, ↑CD4^+^ T cells	Huh-7, HepG2	Yin et al. (2022)
		↑miR-4726-5p, ↓MUC1	HepG2, Huh-7	Tang et al. (2022)
	Evodiamine	↑P53, ↑Bax, ↓Bcl2, ↓cyClinB1, ↓cdc2; ↓NOD1	Human hepatoma cell line Hep3B and Huh-7	Guo et al. (2018)
		↓PRAME	Human hepatoma cell line HepG2	Zhu et al. (2019)
	Matrine	↑Mst1-JNK, ↑JNK phosphorylation, ↑Mst1	Huh-7 liver cancer cell line and HepG2	Cao et al. (2019)
		↓ERK1/2	HepG2	Yu et al. (2020)
		↓EMT	Human hepatoma cell line Huh-7	Wang et al. (2018b)
	Capsaicin	Inhibits the proliferation, migration and invasion of hepatoma cells, hinders cell-matrix adhesion	Human normal liver L02 cells, human hepatoma cells SMMC7721, human hepatoma cell line HepG2	Wang et al. (2019c)
		↓SIRT1, ↓SOX2	WB-F344 cells and HepG2 hepatoma cells	Xie et al. (2022)
		↓SIRT1, ↑NOX4	HepG2 and HL-7702 cells	Hacioglu, (2022)
Phenols	Resveratrol	↑p53; ↓Phosphoinositide 3-kinase/Akt	HCC MHC 97-H cells	Zhang et al. (2018b)
		↑IFN-γ- CD8^+^ T cells, TNF-αand IFN-γ; ↓ STAT3 ↓ Tregs, M2-macrophage; ↓TGF-β1, IL-10	Mouse subcutaneous HCC tumor model	Zhang et al. (2020a)
		↑GST, GPx; ↑CAT、SIRT1; ↓GST-pi, SGPT, SGOT, LPO, NF-κB	A rat model of liver cancer induced by alcohol-aflatoxin B1	Rawat et al. (2021)
		↑SOD, CAT; ↓ sirtuin 1, Urea, MDA	A rat model of liver cancer induced by alcohol-aflatoxin B1	Rawat et al. (2020)
	Curcumin	↓ HSP70; ↓ TLR4, NF-κB	HepG2 heat stress model	Ren et al. (2018)
		↓ IL-6, IL-6R. STAT3, snail, survivin, cyclin D1	A subchronic cell model of TCE induction *in vitro*	Cao and Wang., 2021
		↑caspase-3; ↓ DJ-1, PTEN/PI3K/AKT	HL-7702、SMMC-7721, HepG2	Han et al. (2020)
	Rosmarinic acid	↑caspase-3 and 9、Bax; ↓ Bcl-2	HepG2	Jin et al. (2020a)
		↑caspase-3, Bax; ↓ Bcl-2, PI3K, p-Akt, NF-κB	HepG2	An et al. (2021)
		↑EMT; ↓P13K/AKT/mTOR	SMMC-7721 human hepatoma cells	Wang et al. (2019d)
	Baicalin	↑PTEN, E-cadherin; ↓PI3K/Akt/NF-κB; ↓N-cadherin, MMP-2、MMP-9	Human hepatoma cell lines HepG2, Huh-7; human normal keratinocyte line	Ha et al. (2021)
	Erianin	↓Akt, ERK and P38 phosphorylation; ↓ MMP-2, MMP-7, MMP-9	Human liver cancer SMMC-7721, HepG2	Yang et al. (2020)
	Lysionotin	↑ROS; ↓MMP	HepG2, MMC-7721 cells	Yang et al. (2021)
	Camellia oleifera phenol	↑SHP-1, ↓ JAK1, JAK2, STAT3, EMT	liver cancer cell line LO2, HCCLM3 and Huh7, liver cancer mouse model	Pei et al. (2016)
Polypeptide	ONX0912	↑LC3-II↑protein levels of PINK1 and Parkin, ↑Parkin/Pink pathway induces mitophagy and expands liver cancer cells↓	Human liver cancer cell lines, Huh7, HepG2, 97L, SMMC-7701, SMMC-7721, LM6 and Hep3B	Wu et al. (2021)
	ULK1 inhibitor	↓Expression of FOXM1 and its transcriptional targets, ↓Hepatoma cell proliferation	HepG2	Rajak et al. (2020)
	NMTP-5	↓Endogenous MDM2 protein binds to p53 protein, ↑p53 signal	SK-Hep-1 Cells	Zhou et al. (2021)
	P18 peptide	↓Phosphorylation of VEGFR2, ↓Activation of the PI3K/Akt cascade, ↑mitochondrial-mediated apoptosis and antiangiogenic activity	Human umbilical vein and micro-vascular endothelial cells (HuVECs) and human HCC cell, HepG2	Wang et al. (2017)
Active bacteria/fungi	ZHD-0501	↓Proliferation of human hepatoma cells	Human hepatoma cells	Aly et al. (2021)
	Halomonas sp	↑Apoptosis, ↓G2/M cell cycle	Human hepatoma cell line HepG2	El-Garawani et al. (2020)
	*Lobophorin C*, *Lobophorin D*	cytotoxic activity against liver cancer cells	Human hepatoma cell line HepG2	Bittner et al. (2021)
	*Proteus mirabilis*	↓or kill HepG2 cells	Human hepatoma cell line HepG2	Ren et al. (2021b)
	Spectinabilin ^(1)^	↓Protein levels of cyclin B1 and cdc2; ↓Cell cycle in G2/M phase of SMMC7721 and HepG2 cells	Human hepatoma cell line HepG2	Gao et al. (2019b)

### 3.1 Polysaccharides

Polysaccharides are polymeric carbohydrates composed of 10 or more monosaccharides linked by glycosidic bonds. They are common in animals, plants, microorganisms, and other organisms. Because of their low toxicity and side effects, they are widely used in the food and medicine industries (Zhang et al., 2021). Polysaccharides have a wide range of biological functions. They are used as a source of energy, are a basic component of the body, and also participate in the process of recognition between cells, the regulation of energy supply to the immune system, the transport of intercellular substances, the transformation of cells, and the apoptosis of tumor cells. Research on the antitumor activity of polysaccharides has been the focus of increasing attention. The anti-tumor mechanisms of some plant, animal, marine and fungal polysaccharides are described below according to the source.

#### 3.1.1 Plant polysaccharides

The liver is important for storing iron in the human body, and several studies have shown that iron overload in the liver is positively related to the risk of HCC. Hepcidin (Hepc) is a regulator of iron metabolism. Hepc inhibits the transport of iron ions by binding to membrane iron transport proteins (FPN) located on the plasma membrane of cells (macrophages) on the outer basal side of the intestinal gut. Transporters are eventually degraded in lysosomes, preventing the export of iron ions, which are sequestered in the cell. Ren et al. found that 200 mg/L of dandelion polysaccharide (DP) significantly reduced the expression levels of Hepc protein and mRNA in hepatoma cells and proved that the mechanism of action may be related to inhibition of the JAK-STAT signaling pathway induced by IL-6 (Ren F. et al., 2021). Basil polysaccharide (BPS) extracted from *Ocimum basilicum L.*, which has both pharmacologic and nutrient properties, has been found to induce epithelial-mesenchymal transition (EMT) by blocking hypoxia-inducible factor-1α (HIF1α) under hypoxia, thus producing an anti-metastatic effect. *In vivo* and *in vitro* experiments (with the hepatoma cell line MHCC97H) also proved that BPS increased mRNA and protein levels for the epithelial markers E-cadherin and vesicular protein 1(VMP1), while inhibiting mRNA and protein levels for the interstitial markers N-cadherin (CDH2), vimentin and β-catenin (β-catenin). BPS offers a novel approach for the clinical treatment of malignant tumor metastasis and invasion caused by hypoxia (Feng B. et al., 2019). Alkali-soluble polysaccharide (ALP) and water-soluble polysaccharide (WAP), isolated from the leaves of *Aloe arborescens,* showed direct toxicity against HepG2. WAP can enhance phagocytosis by macrophages, while ALP enhances the activation of lymphocytes to increase the release of cytokines such as interleukin -2 (IL-2), interleukin -12 (IL-12), interferon-gamma (IFN-γ) and TNF-α, which together induce the necrosis and apoptosis of tumor cells (Nazeam et al., 2017).

#### 3.1.2 Animal polysaccharides

Su et al. extracted a new type of polysaccharide (SNP) from *Sipunculus nudus*, and the anti-tumor mechanism of SNP was also investigated. The mechanism of action of SNP is complex, and may include the following: 1. Up-regulation of the expression levels of cytokines in serum, such as IL-2, IFN-γ and TNF-α; 2. Up-regulation of the protein kinase R-like endoplasmic reticulum kinase (PERK)/eukaryotic initiator 2α(elF2α)/activated transcription factor 4 (ATF4)/transcription factor C/EBP homologous protein (CHOP) axis, with a subsequent increase in the mRNA and protein expression levels of cell DNA damage-inducing transcript 3 (DDIT3) in tumors; 3. Reduction in the gene expression levels of cysteine-rich angiogenesis inducer 61(Cyr61), heat shock protein 90(Hsp90) and vascular endothelial growth factor (VEGF) in a dose-dependent manner; 4. Up-regulation of the expression levels of the pro-apoptotic-related proteins caspase-3, caspase-8, caspase-9 and Bax, which induce the apoptosis of tumor cells (Su et al., 2021).

#### 3.1.3 Marine polysaccharides

Extracellular polysaccharide 11 (EPS11) is a natural polysaccharide obtained from the marine bacterium *Bacillus sp. 11*, which has shown remarkable anti-metastatic properties. The extracellular matrix (ECM) is an important component of the tumor microenvironment and is closely related to tumor behavior, including tumor growth, angiogenesis, and metastasis. (Liu X. et al., 2022). proved that EPS11 can directly target type I collagen (an ECM protein) through the β1-integrin signaling pathway (the receptor that mediates ECM-cell interactions), effectively inhibiting the migration of liver cancer cells (Liu et al., 2021a). This research team also confirmed that the filamentous structure promoting cell adhesion was almost completely inhibited when the tumor cells were treated with a 9 nM concentration of EPS11, and that tumor cell migration was reduced under the same conditions. At the same time, mRNA and protein levels for CD99 (a glycosylated transmembrane protein) decreased in a dose-dependent manner, further confirming the anti-cell migration effects of EPS11 (Wang J. et al., 2019). Surprisingly, this research group also isolated and purified EPS364 from *Vibrio alginolyticus 364*, and not only confirmed that EPS364 showed anti-tumor effects that were similar to those of EPS11, but also that it possibly down-regulated the fibroblast growth factor 19 (FGF19)- fibroblast growth factor receptor 4 (FGFR4) signal axis. In this regard, expression of intermediate regulatory molecules such as β-lotho (KLB), β-catenin (CTNNB1), CDH2, alpha-fetoprotein (AFP), activated leukocyte adhesion molecule (ALCAM) and intercellular adhesion molecule-1 (ICAM-1) can induce ROS production and apoptosis, inhibiting the growth and adhesion of cancer cells (Wang et al., 2021a).

Alginate is a polysaccharide rich in sulfate groups which is mainly found in brown algae (Li J. et al., 2020). It has been reported that fucoidan (OF) can bind to the asialoglycoprotein receptor (ASGR) in liver cancer cells, promoting the binding of transcription activator 3 (STAT3) to the P1 promoter of hepatocyte nuclear factor 4A (HNF4A), inducing the expression of P1-HNF4A, and inhibiting the proliferation of cancer cells (Wu et al., 2020). Wang et al. proved that N-dihydrogalactochitosan combined with high-dose X-rays (6–10 Gy) can increase the sensitivity of liver metastatic 4T1_L_3R tumor cells to radiation and significantly increase the damage to DNA. This result shows that the synergistic effect of polysaccharides and ionizing radiation can be used to improve the treatment against metastatic tumor cells (Wang C. Y. et al., 2019).

#### 3.1.4 Fungal polysaccharides

Water-soluble yeast β-D- glucan (WSG) is a polysaccharide composed of D-glucose monomers that naturally exists in bacteria, fungi, algae, and grains. Wang’s research group found that WSG can increase the pH in lysosomes and inhibit cathepsin activity, resulting in dysfunction of lysosomes, blockade of autophagy, and accumulation of damaged mitochondria and ROS, accelerating the death of tumor cells. In addition, WSG reduced the metabolites needed for recycling during glycolysis and the TCA cycle, making HCC cells more sensitive to apoptosis in case of nutritional deficiency. At the same time, this research group also confirmed that WSG can inhibit the growth of mouse primary HCC cells induced by diethylnitrosamine/carbon tetrachloride (DEN/CCl4), without showing signs of toxicity in mice (Wang N. et al., 2020).

Ganoderma lucidum polysaccharide (GLSP) has also been found to exhibit anti-tumor properties, and its mechanisms of action are multifaceted. (Song et al., 2022).found that GLSP-treated macrophages could induce cell cycle arrest in the G2/M phase of hepatocellular carcinoma cells and damage their DNA. Culturing macrophages with GLSP also affected the ratio of classically activated (M1 type) and selectively activated (M2 type) macrophages, and GLSP participated in the inhibition of the PI3K/AKT pathway with phosphatidylinositol kinase and serine/threonine kinase activities. The expression levels of pro-apoptotic factors (Bax, Bad, Bak and Noxa) and of molecules downstream of Bcl-2 were increased, affecting the mitochondrial apoptosis pathway and jointly promoting the apoptosis of tumor cells (Song et al., 2021). Dictyophora polysaccharide has been shown to exhibit potential value for the treatment of liver cancer. After 24 h of treatment with 0.5 mg/ml of Dictyophora polysaccharide, the cell growth inhibition rate was 3.13%. After 24, 48 and 72 h of treatment with 4 mg/ml of Dictyophora polysaccharide, the growth rates of liver cancer cells were inhibited by 45.3%, 59% and 63.4% respectively. This data show that Dictyophora polysaccharide inhibits the growth of liver cancer cells in a dose- and time-dependent manner. Like other polysaccharide inhibitory mechanisms mentioned before, Dictyophora polysaccharide also blocks the cell cycle in the G2/M phase and regulates the expression of pro-apoptotic proteins and genes (Hu et al., 2020).

### 3.2 Alkaloids

Alkaloids are a class of nitrogen-containing alkaline organic compounds that exist in nature and have a variety of biological activities. At present, natural alkaloids with anti-liver cancer properties can be divided into various categories, such as isoquinoline alkaloids, steroidal alkaloids, indole alkaloids and others, according to their different structures.

#### 3.2.1 Isoquinoline alkaloids

Berberine (BBR) is an isoquinoline alkaloid isolated from *Coptis chinensis* Franch. (Zhu et al., 2022). BBR can inhibit the growth of liver cancer cells, and regulate tumor-related pathways to induce apoptosis. Glutamic-pyruvic transaminase 1 (GPT1) is an important regulator of hepatocellular carcinoma growth and plays an important role in amino acid and glucose metabolism. BBR can inhibit the growth of hepatocellular carcinoma cells by inhibiting GPT1 (Guo P. et al., 2020). At the same time, BBR can induce G0/G1 cell cycle arrest in hepatoma cells. Studies have found that BBR can inhibit the Akt pathway and the S-phase kinase-associated protein 2 (Skp2), promote the expression and translocation of Forkhead box O3a (FoxO3a) to the nucleus, and promote the transcription of the cyclin-dependent kinase inhibitors (CDKIs) p21^Cip1^ and p27^Kip1^, resulting in G0/G1 cell cycle arrest in liver cancer cells (Li et al., 2018). Cyclic guanosine 3′,5′-monophosphate (cGMP) is an important signaling molecule downstream of 67LR and plays a key role in cell proliferation, differentiation, and apoptosis. BBR can induce the activation of 67LR, leading to up-regulation of cGMP and activation of caspase-8/3, which ultimately triggers apoptosis (Zhou et al., 2018).

Tetrandrine (Tet) is a natural product isolated and purified from *Stephania tetrandra* S that belongs to the bisbenzylisoquinoline alkaloid family (Luan et al., 2020). Tetrandrine can affect the proliferation and survival of cancer cells and inhibit tumor invasion and migration. Tetrandrine can inhibit the metastasis of hepatoma cells by inhibiting the EMT of human hepatoma cells. In addition, it increases p-β-catenin protein levels and inhibits the Wnt/β-catenin signaling pathway, which is critical for tumor metastasis and normal development (Zhang Z. et al., 2018). CaMKII is a multifunctional serine/threonine kinase that regulates many key biological processes in cancer, including cell division, proliferation, and differentiation. Tetrandrine can significantly reduce the phosphorylation of Ca^2+^/calmodulin-dependent protein kinase II δ (CaMKIIδ), thereby inhibiting its kinase activity and inhibiting the growth of hepatoma cells (Huang et al., 2019).

Lycorine is an alkaloid isolated from *Lycoris radiata*. Lycorine not only induces G2/M cell cycle arrest in HepG2 cells by downregulating cyclin A, cyclin B1 and cell division cycle gene 2 (cdc2), but also inhibits the proliferation and migration of HepG2 hepatoblastoma cells by inhibiting Rho-associated coiled-coil containing protein kinase 1 (ROCK1)/cofilin-induced actin dynamics (Liu et al., 2019). In addition, cyclin-dependent kinase 1 (CDK1) can play an important role in cell cycle regulation and is closely related to the occurrence and development of tumors. Studies have found that lycorine can interfere with the expression of CDK1 to promote senescence and autophagy in liver cancer cells (Yin et al., 2021).

In addition to the above, neferine, extracted from lotus (*Nelumbo nucifera*) seed embryos and colchicine extracted from *Colchicum autumnale* L. also show positive effects in the treatment of liver cancer (Deng et al., 2017; Lin et al., 2021).

#### 3.2.2 Steroidal alkaloids

Solamargine (SM) is a steroidal alkaloid extracted from *Solanum nigrum* Linn. (Kalalinia and Karimi-Sani, 2017). SM can induce apoptosis and autophagy of liver cancer cells and regulate the immune microenvironment. SM can down-regulate the abnormally elevated oncogene LIF in liver cancer tissue and be used to treat liver cancer by inducing autophagy and apoptosis through the LIF/miR192-5p/CyR61/Akt axis. In addition, SM can influence immune cell populations in the immune (tumor) microenvironment by regulating macrophages (Yin et al., 2022). SM also has a significant inhibitory effect on the growth of liver cancer. Studies have found that MUC1 is a key target of SM to inhibit the growth of liver cancer, and that SM can inhibit the growth of liver cancer cells by up-regulating the expression of miR-4726-5p and binding to the MUC1 protein (Tang et al., 2022).

Solanine is a steroidal alkaloid extracted from *Solanum tuberosum* Linn. Studies have found that solanine can enhance the anti-tumor immune response by down-regulating CD4^+^CD25^+^Tregs in tumor tissue and down-regulating the expression of Foxp3 and TGF-β (Gao et al., 2020).

Veratramine is a steroidal alkaloid extracted from *Veratrum nigrum* L. Veratridine not only inhibits the proliferation, migration, and invasion of HepG2 cells, but also induces autophagy and apoptosis. The Bcl2/Bax ratio is considered a decisive factor to determine whether a cell will undergo apoptosis. Veratridine not only significantly up-regulates the expression of Bax and down-regulates the expression of Bcl2, but also significantly up-regulates Beclin-1 and Lc3-II proteins, which are markers of autophagy (Yin et al., 2020).

#### 3.2.3 Indole alkaloids

Evodiamine (Evo) is an indole alkaloid isolated from *Evodia rutaecarpa* Bentham (Yun et al., 2022). Evo not only inhibits the growth of liver cancer cells, but also regulates related signaling pathways to induce apoptosis. Evo up-regulates P53, Bcl2 and Bax; and down-regulates Bcl2, cyClinB1 and cdc2 proteins, significantly inducing G2/M cell cycle arrest in HepG2 cells, and inducing hepatocellular carcinoma cell apoptosis by inhibiting the NOD1 signaling pathway (Guo et al., 2018). In addition, Evo can inhibit tumor metastasis. Melanoma preferentially expressed antigen (PRAME) is highly expressed in patients with liver cancer, and studies have found that Evo can inhibit tumor metastasis by inhibiting the expression of PRAME (Zhu et al., 2019).

In addition, brucine (Qin et al., 2018) and vincristine (Li M. et al., 2021), which are indole alkaloids, have also shown inhibitory effects on the growth and proliferation of tumor cells.

#### 3.2.4 Other alkaloids

Matrine is a piperidine alkaloid isolated from *Sophora flavescens* Ait. Matrine can promote apoptosis of liver cancer cells. The Mst1-JNK pathway is a regulator of mitochondrial homeostasis in metastatic liver cancer cells, and matrine can activate the Mst1-JNK pathway, significantly upregulating JNK phosphorylation and Mst1 expression, leading to mitochondrial fission and apoptosis of liver cancer cells (Cao et al., 2019). Matrine can also inhibit the proliferation and migration of liver cancer cells by regulating related signaling pathways. Extracellular signal-regulated kinases (ERKs), members of the MAPK signal transduction family, are widely involved in cell proliferation and differentiation and in the regulation of growth factor receptors. Studies have found that matrine can inhibit the proliferation and migration of liver cancer cells by downregulating the ERK1/2 signaling pathway (Yu et al., 2020). In addition, matrine can inhibit liver cancer cell invasion and migration by modulating EMT (Wang et al., 2018b).

Capsaicin is a vanillamide alkaloid derived from chili peppers. Capsaicin has shown potential anti-metastatic effects, significantly inhibiting proliferation, migration and invasion by hepatoma cells and hindering cell adhesion to the matrix (Wang K. X. et al., 2019). SOX2 is a transcription factor that maintains the stemness of cancer stem cells (CSCs). Silent information regulator 1 (SIRT1) is a NAD-dependent deacetylase that promotes tumor development. Studies have found that capsaicin can inhibit the occurrence of liver cancer through the SIRT1/SOX2 signaling pathway (Xie et al., 2022). In addition, capsaicin can also affect the SIRT1/NOX4 signaling pathway by reducing the level of SIRT1 protein, increasing NOX4 protein levels and caspase-3/-7 activity, and promoting oxidation, apoptosis and DNA damage in liver cancer cells (Hacioglu, 2022).

Piperine is a pyridine alkaloid isolated from *Piper longum* L. or *Piper nigrum* L. (Yadav et al., 2021). Studies have found that piperine shows potential anti-proliferative effects on CD44^+^/CD133^+^ cancer stem cells isolated from HepG2 cells. It can induce cell cycle arrest in the G1/G0 phase, affecting cell cycle progression (Tiwari et al., 2021).

Dehydrocrenatidine is a β-carolin alkaloid isolated from *Picrasma quassioides*. Dehydroclatidine can induce cell cycle arrest in the G2/M phase, activate the apoptosis pathway mediated by mitochondria and death receptors, reduce the viability of liver cancer cells, and trigger apoptosis of liver cancer cells by inhibiting JNK1/2 phosphorylation (Velmurugan et al., 2022).

Paclitaxel (PTX) is a terpenoid alkaloid isolated from *Taxus brevifolia*. Studies have shown that PTX not only significantly inhibits the proliferation of hepatoma cells, but also inhibits the viability of HLE, L-02 and Bel 7402 hepatoma cells in a time- and dose-dependent manner (Zhu et al., 2016).

### 3.3 Phenols

Phenols are widely found in plants and have a variety of health-promoting effects. Various flavonoids, such as resveratrol, curcumin, rosmarinic acid, baicalin, quercetin, kaempferol, silybin, baicalein, galangin, and luteolin have anti-cancer effects through mechanisms that include scavenging free radicals and inducing cell death (Baby et al., 2021).

#### 3.3.1 Resveratrol

Resveratrol (RES), an important phytochemical component of grapes, is a polyphenol with effects against various types of tumors, including liver cancer, breast cancer, cervical cancer, blood cancer, skin cancer, etc. (Rauf et al., 2018). RES affects various stages, including cancer initiation, promotion, and progression, by modulating multiple signal transduction pathways that control cell growth, division, inflammation, apoptosis, metastasis, and angiogenesis (Ko et al., 2017).

RES can significantly inhibit the viability of hepatoma cells in a time- and dose-dependent manner. RES inhibits the proliferation and migration of hepatoma cells by activating p53 and inhibiting phosphoinositide 3-kinase/Akt-induced autophagy. Combining RES with an autophagy inducer can enhance its antitumor effects (Zhang B. et al., 2018). A study showed that RES inhibited STAT3 signaling and tumor growth in a subcutaneous model of Hepa1-6 liver cancer, reduced the frequency of CD8^+^CD122^+^ Treg and M2-macrophages in the lymph nodes and spleen of tumor-bearing mice, inhibited CD8^+^CD122^+^ Treg CD8 *in vitro* differentiation of CD122^−^ T cells, down-regulated TGF-β1 and interleukin-10 levels in tumors, increased the proportion of IFN-γ-CD8^+^ T cells in tumors and peripheral blood lymphoid organs, and elevated TNF-α and IFN-γ (Zhang Q. et al., 2020).

RES has shown a significant protective effect against alcohol-aflatoxin B1-induced liver cancer. RES significantly reduced GST-pi levels, increased glutathione S-transferase (GST) and glutathione peroxidase (GPx) levels, and enhanced catalase in an alcohol-aflatoxin B1-induced liver cancer rat model. It decreased the levels of liver dysfunction biomarkers (SGPT and SGOT), lipid peroxidation (LPO), and stimulated SIRT1 to inhibit NF-κB, thereby increasing the sensitivity of liver cancer cells to TNF-α-induced apoptosis (Rawat et al., 2021). MSc et al. studied the role of RES and nicotinamide in renal toxicity during alcohol-aflatoxin-B1-induced HCC. It was found that RES treatment normalized urea, lipid peroxidation, lactate, and lactate dehydrogenase levels in hepatocytes, and downregulated elevated SIRT1 expression in hepatocellular carcinoma kidney tissue (Rawat et al., 2020).

#### 3.3.2 Curcumin

The yellow pigment curcumin is an active substance extracted from turmeric. Curcumin (CUR) includes demethoxycurcumin, double demethoxycurcumin and cyclocurcumin. It has shown antioxidant, anti-inflammatory, anti-cancer, anti-diabetic, hepatoprotective, and anti-allergic properties (Dei Cas and Ghidoni, 2019). CUR is widely used to treat a variety of diseases, including lung, cervical, prostate, breast, bone, and liver cancer (Feng et al., 2017).

CUR can regulate the NF-κB pathway, reducing oxidative stress, inflammation, and apoptosis to prevent liver injury (Li W. et al., 2021). It can also activate the p38MAPK pathway, promoting HepG2 cell apoptosis and inhibiting liver tumor growth (He et al., 2021). Upon binding of heat shock protein 70 (HSP70) to Toll-like receptor 4 (TLR4), NF-κB is activated to promote the transcription of inflammatory genes, including cytokines, chemokines, and growth factors. Ren et al. used CUR to treat thermal tolerance HepG2 cells (HepG2TT), and found that CUR inhibited proliferation, metastasis, and invasion of HepG2 cells. CUR significantly inhibited activation of the HSP70-mediated TLR4 signaling pathway by reducing the expression of HSP70 in HepG2TT cells. It inhibited the expression of TLR4 in liver tumor cells, thereby inhibiting the NF-κB pathway (Ren et al., 2018). CUR significantly inhibited IL-6-induced expression of IL-6R, STAT3, snail, survivin, and cyclin D1 in HepG2 cells, and inhibited IL-6/STAT3 to reduce inflammation and EMT. *In vivo*, CUR inhibited the IL-6/STAT3 signaling pathway to control liver tumorigenesis and size. CUR also inhibits HepG2 cell proliferation *in vitro* (Cao et al., 2021). CUR reduced the expression of CXCR4 in PGCs *in vitro* and *in vivo*, thus inhibiting hepatic metastasis of PGCs, possibly by inhibiting stromal cell-derived factor-1/CXCR4 signaling (Gu et al., 2019).

Phosphatase and tensin homologue deleted on chromosome ten (PTEN) exerts tumor suppressor effects by inhibiting the PI3K/AKT signaling pathway, while oncogene DJ-1 can negatively regulate the expression of PTEN. CUR treatment can significantly inhibit the proliferation of SMMC-7721 and HepG2 hepatoma cells, increase the activity of caspase-3, and inhibit the PTEN/PI3K/AKT signaling pathway by downregulating the expression of DJ-1 (Han et al., 2020).

#### 3.3.3 Rosmarinic acid

Rosmarinic acid (RA) significantly reduced the viability of human HepG2 hepatoma cells in a dose-dependent manner, induced apoptosis, activated caspases-3 and 9, and inhibited the migration and invasion of hepatoma cells (Jin et al., 2020a). RA can inhibit the expression of Fyn in HepG2 hepatoma cells, as well as the proliferation, migration, and invasion of hepatoma cells, and the expression of matrix metalloproteinase-2 (MMP-2) and matrix metalloproteinase-9 (MMP-9) in a dose-dependent manner. It increased the expression of cleaved caspase-3 and the pro-apoptotic protein Bax, down-regulated the expression of the apoptosis inhibitory protein Bcl-2 in a dose-dependent manner and promoted the apoptosis of liver cancer cells, reducing the expression levels of PI3K, p-Akt, and NF-κB proteins (An et al., 2021).

EMT is a key regulator of tumor invasion and metastasis. RA can inhibit invasion by the hepatoma cell line SMMC-7721 cells by regulating EMT. It also promotes tumor cell apoptosis and inhibits the activation of the P13K/AKT/mTOR signaling pathway *in vitro* and *in vivo* (Wang L. et al., 2019).

#### 3.3.4 Baicalin

Baicalin has shown different degrees of anticancer activity against liver, gastric, non-small cell lung, cervical, and esophageal cancer (Ji et al., 2021). Treatment with baicalin (25, 50 and 100 μM) can inhibit the migration and proliferation of HepG2 cells in a dose-dependent manner. It can also down-regulate PI3K/Akt/NF-κB signaling pathway-related proteins by increasing the expression of PTEN, regulate EMT markers and migration-related proteins, and regulate HepG2 cell migration by upregulating E-cadherin and downregulating of CDH2, MMP-2, and MMP-9 (Ha et al., 2021).

#### 3.3.5 Quercetin

Quercetin inhibits liver inflammation mainly through NF-κB/TLR/NLRP3. It also reduces oxidative stress mediated by PI3K/nrf2, activates mTOR during autophagy, and inhibits the expression of apoptotic factors related to the development of liver disease. In addition, quercetin shows different mechanisms of action at different stages of liver disease, including regulation of PPAR, UCP, and plin2-related factors through brown fat activation in hepatic steatosis. This compound inhibits stromal ECM deposition during the fibrotic liver stage, affecting TGF1β, ERs and apoptosis. In the late stage of liver cancer, it can inhibit the proliferation and spread of cancer cells by modulating the hTERT, MEK1/ERK1/2, Notch, and Wnt/β-catenin related signaling pathways (Zhao et al., 2021).

#### 3.3.6 Others

Tannins play important roles by regulating multiple tumor signaling pathways, including the JAK/STAT, RAS/RAF/mTOR, TGF-β1/TGF-β1R, VEGF/VEGFR and CXCL12/CXCR4 axes. Tannins relieve liver cancer and show antifibrotic and anticancer effects (A. Youness et al., 2021). Erianin can reduce the proliferation, migration and invasion of liver cancer cells; down-regulate invasion-related proteins such as MMP-2, MMP-7, and MMP-9; promote cell apoptosis; and inhibit Akt and ERK/P38 phosphorylation in the PI3K/Akt and ERK/P38 pathways (Yang et al., 2020). Lysionotin, a flavonoid compound found in *Lysionotus pauciflorus* Maxim, can significantly reduce cell viability, inhibit cell proliferation and migration, promote cell apoptosis, increase the levels of intracellular ROS, reduce mitochondrial membrane potential (MMP), and alter the content of apoptosis-related proteins, inhibiting the growth of HepG2 and SMMC-7721 tumors. It also shows significant anti-HCC effects (Yang et al., 2021). Oleocanthal, a phenolic compound found in olive oil, exerts its anti-hepatocellular carcinoma effects by reducing the activities of JAK1 and JAK2, increasing the activity of SHP-1, inhibiting the activation of STAT3, and inhibiting EMT (Pei et al., 2016). Honey contains polyphenols, such as chrysin, isorhamnetin, myricetin, protocatechuic acid, etc., which can protect against liver disease and hepatocellular carcinoma by regulating NF-κB levels, oxidative stress, inflammation, etc. (Talebi et al., 2020). Daphne flavone has shown significant inhibitory effects against hepatoma cells, and histone deacetylase 6 (HDAC6) has been identified as a potential target of Daphne flavonoid (Chen et al., 2021).

### 3.4 Polypeptides

Polypeptides usually consist of 10–100 amino acids linked by peptide bonds. They have relative molecular weights between 500 and 10,000, which fall in between that of small chemicals and proteins. This low molecular weight can potentially allow them to penetrate tissues. At present, several polypeptide drugs with anti-liver cancer activity have been widely studied.

#### 3.4.1 Protease inhibitors

Protease inhibitors are polypeptide compounds showing anti-tumor effects. Protease inhibitors play active roles in anti-liver cancer therapies by regulating signaling pathways which induce apoptosis and autophagy of liver cancer cells. They can also inhibit the growth of liver cancer cells.

ONX0912 is a novel oral proteasome inhibitor. ONX0912 can be used to treat liver cancer since it promotes mitochondrial autophagy and apoptosis. ONX0912 can up-regulate PINK1 and Parkin protein expression levels, subsequently activating the Parkin/Pink pathway to induce mitochondrial autophagy. In addition, ONX0912 also induces collapse of the mitochondrial membrane potential and increases mitochondrial ROS levels in tumor cells, triggering apoptosis through the intrinsic mitochondrial pathway (Wu et al., 2021). Unc-51 Like Autophagy Activating Kinase 1(ULK1) inhibitor can inhibit autophagy in liver cancer cells. FoxM1 is associated with proliferation, clone formation, drug resistance, anti-apoptosis, cell cycle and senescence induction in hepatoma cells (Hu et al., 2019). Studies have shown that the autophagy protein ULK1 inhibitor attenuates the expression of FOXM1 and its transcriptional targets, and also shows a synergistic effect when combined with FOXM1 inhibitor to inhibit the growth of HepG2 (Rajak et al., 2020).

#### 3.4.2 Other polypeptides

Anticancer peptides (ACPs) are bioactive peptides extracted from antibacterial peptides (AMPs) or natural resources, which are widely found in many organisms, including mammals, amphibians, insects, plants, and microorganisms. ACPs can show anti-tumor effects through immunomodulation. Tumor-associated antigens (TAAs) can be presented by antigen-presenting cells to induce the activation of tumor-responsive T lymphocytes. ACPs derived from TAA have shown immunostimulant activity. They can be used to effectively target tumor cells within an immunosuppressive microenvironment, and play a significant role in enhancing anti-HCC therapy (Zhang C. et al., 2019). SP94 (SFSIIHTPILPL) is a peptide isolated using phage-displayed selection. Related studies have shown that pegylated doxorubicin liposome (SP94-LD), formed by coupling the SP94 peptide with non-targeted pegylated liposomal doxorubicin (LD), can inhibit the growth of human hepatocellular carcinoma xenografts without showing toxicity against normal cells (Wu et al., 2018). NMTP-5 is a kind of NRP1/MDM2-targeted d-peptide supramolecular nanomedicine. Relevant studies have shown that MDM2 is a negative regulator of p53. NMTP-5 can activate p53 signaling by targeting NRP1 into the cytoplasm of cancer cells, and at the same time interfere with MDM2-p53 interaction to up-regulate p53 levels and activate the expression of target genes that mediate cell cycle arrest and apoptosis in hepatocellular carcinoma (Zhou et al., 2021).

Pigment epithelial-derived factor (PEDF) is an endogenous angiogenesis inhibitor. A short and stable functional peptide, P18, extracted from PEDF can enhance its stability to inhibit angiogenesis more effectively. Relevant studies have shown that the P18 peptide can target the phosphorylation of endothelial growth factor receptor 2 (VEGFR2), modulate signaling transduction between VEGF and VEGFR2 and suppress activation of the PI3K/Akt cascade, leading to an increase in mitochondrial-mediated apoptosis and anti-angiogenic activity (Wang et al., 2017). The C7 peptide is a candidate peptide composed of 7 amino acids, which has moderate affinity and good specificity towards the mesenchymal-epithelial transition (c-Met) on the surface of phages. C7 peptide can prevent metastasis and invasion in liver cancer by inhibiting the Akt and Erk1/2 signaling pathway, blocking the combination of HGF and c-Met, and inhibiting HGF/c-Met and its downstream signaling pathway (Zhao et al., 2019b).

### 3.5 Active bacteria/fungi

Currently, natural product extracts are the most promising source of new anticancer drugs. Bacteria are the largest producers of biologically active natural products and are of enormous importance for drug discovery (El-Garawani et al., 2020). Some crude extracts exhibit selective anticancer activity against specific tumor cell lines, while having no effect on non-tumor cell lines. Studies have shown that strains exhibiting cytotoxic activity (Micromonas, *Streptomyces*, Actinomycetes, etc.) are known producers of anticancer compounds. Related studies have shown that the *Protobacterium luteum* MOSEL-ME10a, found in the ocean, shows a significant inhibitory effect against HepG2 cells (Tanveer et al., 2021). Actinomycete species extracted from marine sediments have also been described to produce metabolites with potent anticancer activity, such as ZHD-0501, a species that has been shown to inhibit the proliferation of human liver cancer and leukemia cells (Aly et al., 2021). *Lobophorin C* and *Lobophorin D* were isolated from the fermentation broth of the symbiotic actinomycete *Streptomyces carnosus* strain AZS 17, associated with the sponge *Hymeniacidon sp*. in the coastal waters of the East China Sea, and from sediment samples from the Cantabrian Sea (at a depth of 2000 m by Sarmiento-Vizcaíno et al.). In addition, *paulomycin G* produced by the marine strain *matsumotoense m-412,* exhibited cytotoxicity against human hepatoma HepG2 cells (Bittner et al., 2021).

When HepG2 cells were treated with the ocean-dwelling *Halomonas sp*. (GWS-BW-H8hM strain), it was also found that it exerted anticancer effects by inhibiting apoptosis initiation and cell cycle progression. Studies showed that the Bax/BCL-2 ratio, based on mRNA expression, was significantly increased, and that caspase-3 and p53 were up-regulated after H. HA1 bacterial extract was applied to HepG2 cells. It is known that Bax is up-regulated, whereas BCL-2 protein expression is down-regulated, by p53 protein (El-Garawani et al., 2020). p53 is a nuclear transcription factor that is normally activated during apoptosis and which regulates a number of downstream effectors (Kanapathipillai, 2018). Apoptosis can be induced in a transcription-independent manner through mitochondrial localization of p53 or by inducing endoplasmic reticulum stress to prevent p53-dependent apoptosis through the glycogen synthase kinase 3β pathway (D’Arcy, 2019). Expression profiling of H. HA1 extracts indicated a p53-dependent mitochondrial apoptotic pathway. *Halomonas sp.* was shown to exert an antiproliferative effect on HepG2 cells (El-Garawani et al., 2020).

Eighteen species of actinomycetes were isolated from three species of Erylus sponges collected from the seas of Portugal, namely *E. discophorus* (Berg01 and Berg02), *E. deficiens* (#91) and *E. mamillaris* (SM). Experiments by Ren et al. showed that extracts (195 μL) from strains Berg02-79 (medium: IN - CRY), Berg02-22.2 (IN - CRY) and #91_40 (IN - CRY) had some inhibitory effect on HepG2 cells. In 2019, Santos et al. tested 12 Firmicutes and 44 Proteobacteria isolated from Erylus spp. sponges obtained from Portuguese waters. Growth media and extraction methods were like those used with Actinobacteria. The extracts from *Proteus mirabilis* #118_13, (IN - CRY), *Pseudovibrio sp.* Berg02_9.1 (IN - CRY) and *Psychrobacter celer* #118_17 (IN - CRY) (5 μL in 195 μL medium) showed the strongest growth inhibitory effect against HepG2 hepatoma cells (Santos et al., 2020). In addition, in the medium-scale fermentation culture screening performed in the EPA vial, 5 μL of Mirabilis (*Proteus mirabilis* #118_13 (IN-CRY), *Paenibacillus sp*. #91_7 (IN-CRY) and *Pseudovibrio sp.* Berg02_10) inhibited or were lethal for HepG2 cells (Ren X. et al., 2021).

Spectinabilin^(1)^, a Spectinabilin derivative^(2)^ and a novel analog, 2-desmethyl-Spectinabilin^(3)^, were isolated from a *Streptomyces spectabilis* fermentation broth. Studies have shown that Spectinabilin ^(1)^ exerts anti-tumor effects against SMMC7721 and HepG2 cells by down-regulating the PI3K/AKT signaling pathway, which is involved in a series of processes such as cell proliferation, tumorigenesis, and development. Further studies have demonstrated that Spectinabilin^(1)^ increases p21 protein levels (a cyclin-dependent kinase inhibitor located downstream of the p53 gene) by reducing cyclin B1 and cdc2 protein levels, triggering G2/M cell cycle in SMMC7721 human hepatoma and HepG2 cells. Spectinabilin ^(1)^ induces apoptosis of SMMC7721 and HepG2 cells by down-regulating Bcl-2 protein expression, up-regulating Bax protein expression, and activating the cleavage of caspase-9 and caspase-3 (Gao X. et al., 2019).

Lactic acid bacteria, including Bifidobacteria, are chemopreventive against colon, bladder, liver, breast, and stomach cancers (Riaz Rajoka et al., 2017). Biotransformation is one of the mechanisms by which Bifidobacteria exert their antitumor effects. Essentially, the biotransformation function is accomplished by converting compounds into usable energy through biological processes. Many Bifidobacteria may be involved in the production of enterolactone, which has antitumor effects. Bifidobacteria also exhibit antitumor effects by altering the expression of cancer-related genes and cytokines (Wei et al., 2018).

## 4 Discussion

As one of the common causes of cancer-related death in the world, liver cancer not only seriously threatens human health, but also brings a huge economic and public health burden to the world (Lv et al., 2020). At present, the treatment of cancer is still mainly surgery, combined with adjuvant therapy such as chemotherapy, radiotherapy, drug-targeted therapy, and immunotherapy. However, surgical resection or liver transplantation also has certain limitations. The low resection rate and high recurrence rate are constraints for liver cancer surgery. For most patients with liver cancer, especially in the advanced stage, non-surgical treatment is still an important treatment method, but it usually brings serious complications to the patients (Dika and Abou-Alfa., 2017). Therefore, exploring a therapeutic approach with curative effect on liver cancer and low side effects has become a new direction for cancer treatment in the future. In this review, we mainly summarize the common pathogenesis of liver cancer (metabolic fatty liver, virus and alcohol) and the role of bioactive substances such as polysaccharides, alkaloids, phenols, polypeptides and active bacteria/fungi in the treatment mechanism of liver cancer. We aim to combine pathogenic and therapeutic mechanisms to provide a certain convenient way for scholars who are studying this aspect, and at the same time expect to propose reliable scientific evidence and more possibilities on the targets and signaling pathways of liver cancer treatment.

At the same time, we also found that there are still some issues that need to be solved in the treatment of liver cancer:

1) Liver cancer has an insidious onset. It lacks typical symptoms in the early stage, and most patients have developed to the middle and late stages when clinical symptoms appear, thus losing the optimal timing of treatment and other difficult-to-control characteristics. How to improve the precision of liver cancer screening should also become the focus of research (Hartke et al., 2017).
2) Tumor metastasis is one of the main causes of death in most cancer patients, and inhibiting metastasis makes it possible to prolong the survival time of cancer patients and even cure cancer (Fan et al., 2017). However, the treatment of cancer metastases is not only related to the secondary tumor, but also to the characteristics of the primary tumor itself and the effectiveness of its available targets (Liu et al., 2022). Therefore, looking for targets related to liver cancer and primary tumor metastasis, understanding the mechanism of action of these markers or signaling pathways, and developing targeted drugs, will open up a new situation for the treatment of liver cancer.
3) The treatment of liver cancer needs to cover multiple targets and formulate an overall treatment strategy. Some of the reported new therapeutic targets have not yet been truly validated and transformed in clinical practice. For example, researches on the tumor immune microenvironment and immunosuppressive therapy of liver cancer still require a lot of preclinical studies (Oura et al., 2021). We believe that in future research, we can not only try to discover new therapeutic pathways, but also try to combine multiple therapeutic pathways to analyze the mechanism of action of bioactive substances in the treatment of liver cancer.
4) In addition, whether various active ingredients with different regulatory effects can play a common role to form an anti-hepatocellular carcinoma interaction network from various aspects such as apoptosis, cycle operation, invasion and metastasis. In the future, it is necessary to further explore the specific targets of active ingredients. Through network pharmacology, genomics, metabolomics and other methods to sort out relevant targets, find the possibility of a variety of active ingredients working together to fight cancer in an all-round way, and provide a more powerful reference for the development of more scientific and effective anti-cancer drugs (Ruan et al., 2022).
5) More toxicity and dose studies are needed for biologically active substances. At present, most studies focus on exploring the therapeutic mechanism of bioactive substances on liver cancer, and studies on the toxicity and related doses of bioactive substances still need to be supplemented to support further clinical research.
6) Novel drug delivery systems such as lipid nanoparticles are promising and efficiently tailored drug delivery systems in liver cancer therapy, which can provide a new avenue for highly specific and efficient liver cancer therapy (Alanazi et al., 2020). For example, Zhang et al. designed a preparation of doxorubicin (DOX) targeted for the treatment of liver cancer, using ASP as a hydrophilic group and deoxycholic acid (DOCA) as a hydrophobic group to form an amphiphilic conjugate (ASP-DOCA), and prepared DOX-loaded chemotherapeutic drug nanoparticles (DOX/ASP-DOCA NPs). In addition to showing higher affinity for the asialoglycoprotein receptor (ASGPR) when compared with free DOX, ASP exerted more significant inhibition of tumor cell proliferation, while reducing the side effects of free DOX (Zhang Y. et al., 2019). At the same time, the combination with the existing hepatocellular carcinoma chemotherapy drugs is also a new direction for future research. Combination therapy with Cur and sorafenib was found to reduce cyclin A, B2 and D1 protein levels, phosphorylated retinoblastoma and B-cell lymphoma (Bcl) super large protein. Sorafenib, Cur or kaempferol (KMF) combination therapy can cause S-phase and G2/M-phase arrest of hepatoma cells and significantly induce apoptosis (Bahman et al., 2018). Furthermore, artificial synthesis is another method to improve the effectiveness of active substances against liver cancer. Peng Zhu et al. designed and synthesized 14 novel phenylallyl cyclohexanone analogs based on piperine. The results showed that the newly synthesized compound 9M could inhibit thioredoxin reductase (TrxR) activity, increase ROS levels, reduce the mitochondrial transmembrane potential (MTP) by regulating autophagy-related proteins Lc3, p62 and Beclin-1, and induce autophagy in Bel7402/5-FU hepatoma cells (Zhu et al., 2020). In future research, more attention should be paid to the fields of bioavailability, pharmacokinetics and pharmacodynamics of biologically active substances, based on the preparation of novel drug delivery systems for use in combination with existing chemotherapeutic drugs for hepatocellular carcinoma, and artificial synthesis of new substances. Explore how more bioactive substances can be used as effective anticancer drugs, bridging the gap between preclinical research and clinical research (Rodriguez et al., 2021).
7) As mentioned earlier, underlying liver diseases (such as hepatitis virus infection, alcohol abuse, or nonalcoholic fatty liver disease) occur in the majority of HCC patients, which means that most HCC cases occur in the context of chronic inflammation. Therefore, linking liver immunology and tumor therapy, and finding the connection and effective targets between the two will help provide opportunities for designing personalized combined immunotherapy for advanced HCC patients (Lindblad et al., 2021).
